# Droplet Coalescence by Selective Wettability Enhancement in Microfluidic Devices

**DOI:** 10.3390/nano10040737

**Published:** 2020-04-12

**Authors:** Nahla Alamoodi, Anas Alazzam

**Affiliations:** 1 Research and Innovation Center on CO_2_ and H_2_ (RICH), Center of Catalysis and Separation (CeCaS), Chemical Engineering Department, Khalifa University of Science and Technology, Abu Dhabi 127788, UAE; 2 System on Chip Center, Mechanical Engineering Department, Khalifa University of Science and Technology, Abu Dhabi 127788, UAE

**Keywords:** graphene oxide, wettability patterning, coalescence, microfluidics, patterned surface energy, microfabrication

## Abstract

A new approach for droplet coalescence in microfluidic channels based on selective surface energy alteration is demonstrated. The proposed method involves patterning the surface of cyclic olefin copolymer (COC), a hydrophobic substrate attached to a polydimethylsiloxane hydrophobic microchannel, with graphene oxide (GO) using standard microfabrication techniques. Surface wettability and adhesion analyses confirmed the enhancement of the COC surface energy upon GO patterning and the stability of the GO film on COC. Three representative cases are illustrated to demonstrate the effectiveness of the method on the coalescence of droplets for different droplet flow regimes, as well as the effect of changing the size of the patterned surface area on the fusion process. The method achieves droplet coalescence without the need for precise synchronization.

## 1. Introduction

Coalescence of droplets in microfluidic systems offers many advantages such as high mixing rates, continuous separation of multiphase systems, and creating reaction-controlled nanoliter-sized individual reactors [1,2,3]. The mechanism of coalescing droplets in a multiphase system where droplets of one phase are dispersed in another continuous phase can be divided into three subprocesses [4]. The first subprocess is the collision of two droplets trapping a thin film of the continuous phase between them, the second is the drainage of the thin film due to the van der Waals force of attraction between the droplets, and the third is overcoming the surface tension of the individual droplets by attraction forces leading to their fusion [5].

Droplets coalescence techniques in microfluidic channels can be classified into active and passive techniques. Active techniques utilize an external field to generate energy that destabilizes the interfaces of adjacent droplets leading to droplet fusion [2,3]. Such techniques include electrocoalescence [6,7], dielectrophoresis (DEP) [8,9], magnetophoretically actuated droplet coalescence [10], and temperature and pneumatically actuated droplet coalescence [11,12,13]. While active techniques result in highly controlled droplet dynamics, they are inherently complex techniques requiring complicated methods for the fabrication of microfluidic channels with integrated elements to be activated by the external sources [14]. Moreover, the presence of external fields limits the use of the active techniques to specific compatible systems. Passive droplets coalescence techniques involve manipulating the dynamics of the different fluids in the system. They generally result in hindering or slowing down the motion of the droplets by a fluid resistant element for the subsequent droplets to approach and collide with them [2]. The fluid resistant element can be created by the channels’ geometrical orientations and introducing microstructures or by surface treatment.

Recent studies of geometrically induced coalescence included inducing droplet coalescence in the merging zone of Y- and T-junction channels [15,16], and by introducing microgrooves [16], micropillars [17], micro-lancet [14], and by microexpansion techniques [18], and microfluidic traps [19]. These techniques are restricted to the design of the channels and require accurate droplet synchronization [3]. Surface induced droplet coalescence methods involve altering the surface energy of the channel by changing its wettability to create a flow resistance as a result of the difference between the drag viscous flow and the surface energy [2]. While there is on-going research on fabricating channels from two materials with different surface energies, there is a single study on the selective patterning of surface energy where the droplet coalescence is passively induced by patterning the surface of a hydrophobic channel with hydrophilic poly (acrylic acid) that is grafted via UV photopolymerization [3].

In the present paper we report a new approach for droplet coalescence based on selective alteration of the surface wettability of the microfluidic channel using graphene oxide (GO). This approach involves the fabrication of a polydimethylsiloxane (PDMS) microchannel on a hydrophobic planar cyclic olefin copolymer (COC) substrate patterned with hydrophilic graphene oxide (GO) using standard microfabrication techniques.

## 2. Materials and Methods

**Figure 1:**
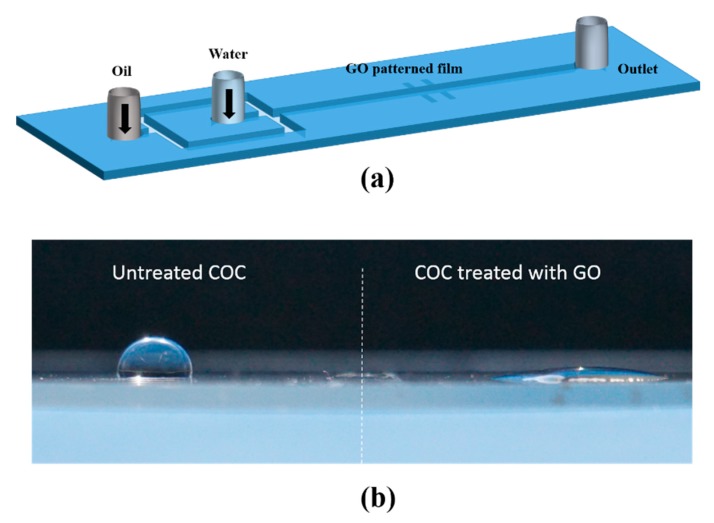
(**a**) A schematic of the Polydimethylsiloxane (PDMS)- Cyclic Olefin Copolymer (COC) microfluidic device with a flow focusing droplet generation scheme and a patterned wettability surface. (**b**) An image of the spreading of a water droplet on a COC substrate treated with graphene oxide (**right**) compared to a water droplet of the same volume standing on untreated COC (**left**).

The patterning of COC substrates (SIRRIS.be, Brussel, Belgium) with GO (Sigma Aldrich, St. Louis, MI, USA) was achieved using plasma-enhanced lift-off method [20,21]. Briefly, the desired GO pattern was first designed in CAD software. Then the COC substrate was coated with a photoresist (Microposit S 1813) using a spin coater (WS650Hzb-23NPP UD-3 from Laurell Technologies Corporation, North Wales, PA, USA). After post baking the substrate for 2 min at 70 °C, the GO design was patterned using a UV photolithography system (Dilase 650 from KLOE) and developed. Next, it was treated with oxygen plasma and coated with GO using a spin coater. Finally, lift-off process was performed in acetone (Fisher Scientifics). The microfluidic device was completed by molding the PDMS (Sylgard 184, Dow Corning) channel, treating it and the GO-coated COC substrate with oxygen plasma, aligning them, and bringing both surfaces together to bond them. Figure 1a is a schematic for the patterned microfluidic device with a flow focusing droplet generation scheme. The device was tested for the coalescence of water droplets colored with a food dye dispersed in silicone oil (10 cP, Sigma Aldrich). For testing, syringe pumps (neMESYS, Cetoni GmbH, Germany) are used to pump the liquids into the channel, and a high-speed camera (Fastcam SA-X2, Photron, Tokya, Japan) and an optical inverted microscope (Zeiss Axio, Carl Zeiss, Oberkochen, Germany) are used for imaging.

## 3. Results and Discussion

The surface energy enhancement of the COC substrate was assessed by measuring the static contact angle of a water droplet on the COC substrate with and without GO deposition. Figure 1b shows the spreading of a water droplet on a COC substrate that is half coated with GO (right) compared to a water droplet of the same volume standing on untreated COC (left). The contact angles measured were 10° and 120°, respectively. Water contact angles for GO measured in a typical method is reported to be 30°–60° [22], lower contact angles have been reported to be due to multiple layers of GO, resulting in an enhanced droplet−GO interaction [23].

**Figure 2:**
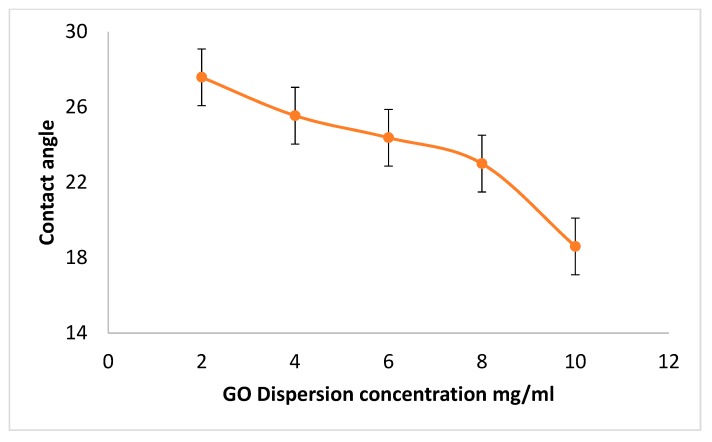
Contact angle of a water droplet on COC wafer coated with different concentrations of GO dispersion.

The surface energy enhancement was further investigated by measuring the contact angle for different concentrations of GO dispersion deposited on COC wafers. Five GO dispersions were prepared with concentrations of 2, 4, 6, 8, and 10 mg/mL, respectively, and were deposited on plasma treated COC wafers using a spin coater at 4000 rpm. Figure 2 illustrates the effect of increasing the GO dispersion concentration on the water contact angle. The decreasing behavior of the contact angles with values of 27.6°, 25.5°, 24.4°, 23°, and 18.6°, respectively, confirms the ability of increasing the surface energy by increasing the concentration of the GO dispersion, and implies the same by using GO with more oxygen-containing functional groups, i.e., GO with lower C/O ratios. The hydrophilicity of the GO film was investigated over a period of three days. No change in the contact angle was observed if the film was not exposed to light or heat, as both have been found to alter hydrophilicity.

**Figure 3:**
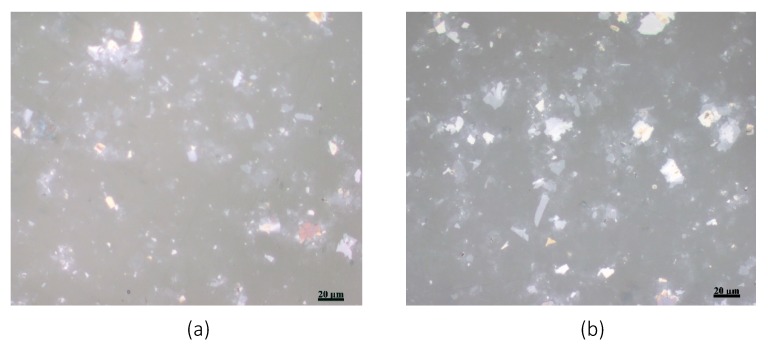
Two microscopic images of the GO film on top of COC wafer (**a**) before and (**b**) after the water boiling test.

The stability of the GO film on the COC wafer was also studied using the JIS K-6744 boiling water test. A COC wafer was coated with GO with a concentration of 4 mg/mL, as discussed above, and was immersed in boiling water for an hour. Optical microscopic images were taken at several locations of the COC wafer before and after the test. Figure 3 shows two microscopic images of the GO-coated COC wafer. Both images, as reflected in this figure, show similar flake distribution without evidence of the GO film peeling off from the COC wafer.

**Figure 4:**
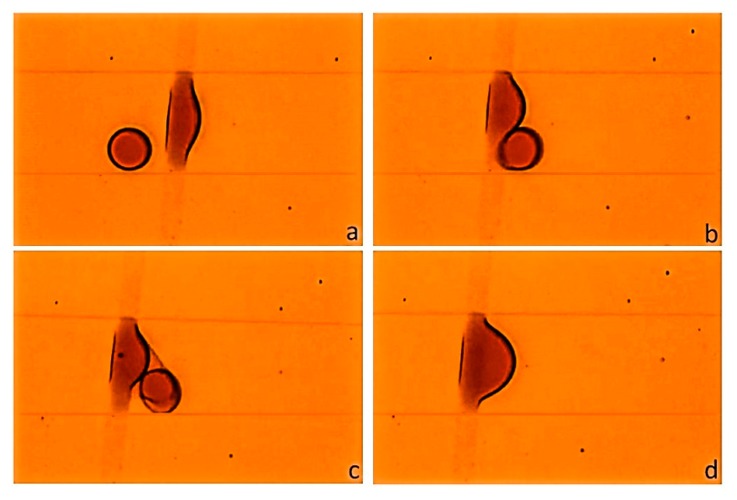
Droplet coalescence process in a microfluidic channel with patterned GO. (**a**) Droplet wetting the GO band, (**b**) subsequent droplet in contact with the droplet wetting the GO surface, (**c**) A bridge was formed between the droplets, (**d**) coalescence of droplets. The dimensions of the channel (W × H) are 200 µm × 50 µm. The width of the GO band is approximately 30 µm.

The ability to selectively enhance the surface energy of COC by GO patterning is used to control the wettability inside microfluidic systems to achieve droplet coalescence. The droplet coalescence mechanism observed in the proposed device is composed of three steps: trapping, fusion, and detachment of the merged composite droplet. The coalescence of water droplets using GO bands that are patterned perpendicular to the flow or with an angle along the channel is investigated. Figure 4 illustrates the trapping and the fusion steps. A 30 µm width GO band is patterned perpendicular to the length of the microchannel in which droplets of dye solution are generated in a continuous phase of silicone oil using a flow-focused configuration. In the trapping step, Figure 4a, where the surface force dominated the viscous drag force, the oxygen-containing functional groups in the GO formed hydrogen bonds with the water molecules in the dye solution [23]. This bonding process caused the water molecules to accumulate until a thin film of dye solution is formed over the GO pattern. Consequently, as a droplet passes across the patterned GO it gets trapped to the thin film. The subsequent droplet then gets in close contact with the trapped one (Figure 4b), separated by a thin film of the continuous phase. Due to the attraction forces between them, the continuous phase film separating the two droplets drains and a thin liquid bridge connecting them is formed (Figure 4c) [2]. The formed bridge resulted in a surface tension imbalance causing the two droplets to coalesce (Figure 4d).

The succeeding droplets continue to coalesce with the merged droplet as long as the adhesion forces, due to the patterned enhanced surface energy of the surface, dominates the viscous drag force of the fluid. As the magnitude of the viscous drag force exceeds the surface forces, the merged droplet detaches from the GO pattern. Several case studies have been conducted to examine the effect of the length and the orientation of the GO pattern on 1) The coalescence of droplets exhibiting different flow regimes, and on 2) The detachment mechanism of the merged droplet. Three representative cases will be illustrated. The first case demonstrates the detachment process of the merged droplet created from disc or pancake-shaped droplets and its relation to the number of coalescing droplets, the second case demonstrates the coalescing of two slug droplets over a narrow band of GO (~10 µm), and the third case illustrates the effect of patterning GO for coalescing droplets and directing the motion of the merged droplet to a specific path.

Disc-shaped droplets are the ones confined between the top and bottom sides of the channel with a size smaller than the width of the channel. Controlling the flow and the coalescence of such droplets permits the creation of microdroplet reactors, where merging droplets of different components is necessary at a selected site in the microchannel.

Theoretically, the detachment of the merged droplet in this regime is initiated as the viscous drag force exerted by the continuous phase overcomes the force due to the enhanced surface energy [24]. In the illustrated case, however, the merged droplet breaks off, leaving behind a residue of the dispersed phase on the GO pattern. This indicates that the adhesion force between the dye solution and the GO is stronger than the viscous drag force experienced by the droplet, as well as the cohesive forces within the droplet. This observation was also reported in a previous study for the detachment of a water droplet from surfaces with contact angle less than 90° [25]. The detachment then occurred as a result of the viscous drag force overcoming the surface tension of the merged droplet, leaving behind a thin film of the dye solution wetting the surface of the patterned GO. The viscous drag force created by the continuous phase produced shear stress on the interface of the droplet causing it to stretch. As the volume of the merged droplet increased due to the coalescence of more droplets, the drag force ($F_{drag}\propto \mu vR,$ where R is the droplet radius, μ is the dynamic viscosity, and is v the continuous flow velocity relative to the dispersed phase [26]) increased and the interface stretched further, resulting in a decrease of the diameter of the droplet near the patterned GO. The diameter near the patterned GO decreased further forming a neck that eventually became a pinch-off or a break-up point for the merged droplet due to capillary (Rayleigh-Plateau) instability.

**Figure 5:**
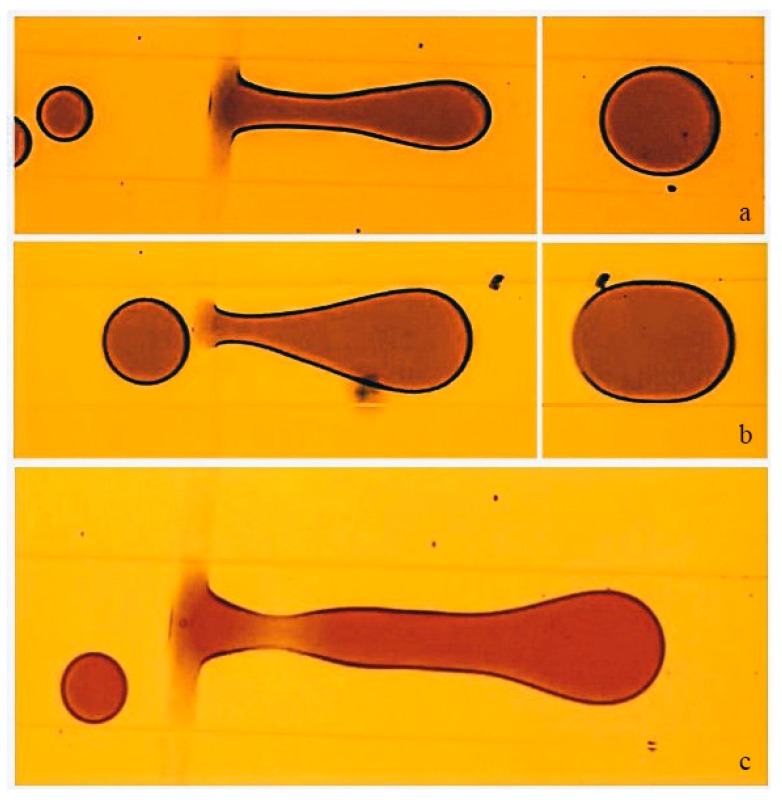
Coalescence and detachment process of merged droplet from the GO patterned COC surface. (**a**) Merging of six droplets with initial diameter of 86 µm, the flow rates were 300 µL/h for oil and 50 µL/h for water (**b**) Merging of three droplets with initial diameter of 138 µm, the flow rates were 250 µL/h for oil and 50 µL/h for water (**c**) Neck development before the detachment of the merged droplet. The dimension of the channel (W × H) is 200 µm × 50 µm. The width of the GO pattern is approximately 30 µm and is patterned along the width of the channel.

From the discussion above, the number of merging droplets depends on the competing forces of the increasing viscous drag force and the surface tension on the merged droplet. For example, an increase in the continuous flow velocity results in merging smaller number of droplets compared to a lower velocity of the continuous phase. Similarly, merging of droplets with large diameters will result in the merging of a fewer number of droplets compared to merging of droplets with smaller diameters. Figure 5 illustrates the droplet coalescence of droplets in two streams having different initial diameters. The merged droplet in Figure 5a shows the merging of six droplets while Figure 5b shows the merging of three droplets. The micrograph in Figure 5c shows the neck that resulted in the pinch-off of the merged droplet.

**Figure 6:**
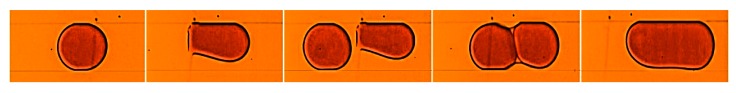
Sequence of trapping, fusion, and detachment of slug droplets over a narrow band of GO. The dimensions of the channel (W × H) are 200 µm × 50 µm. The width of the GO pattern is approximately 10 µm and is patterned along the width of the channel.

The second case demonstrates the coalescing of two slug droplets over a narrow band of GO (~10 µm). Slug droplets are droplets that are confined by the four walls of the channel and that have a length that is mainly affected by the phase flow rate ratio of the inlet streams at the droplet generation stage [1]. In this flow regime circular flows exist in the dispersed droplet slugs and in the surrounding continuous phase [27]. These flows enhance mixing in phases making it effective in enhancing mass transfer between the dispersed and continuous phases for processes such as extraction [1]. The coalescence process of the two slugs is similar to the process described in Figure 4 with a slight variation with respect to trapping and detachment. After the slug droplet is trapped by the GO band, it changes its shape and detaches from the wall of the channel to reduce the viscous drag force exerted on the droplet. As the second slug advances, the drag force became enough to push the trapped slug away from the GO as both slugs collided. Both slugs then remained in close proximity as the thin film of the continuous phase separating them drained leading to their fusion. The merged droplet, having a drag force greater than the forces due to the enhanced surface energy, detaches from the GO pattern and proceeds in the channel. Figure 6 shows a sequence of droplet coalescence over a narrow band of GO.

**Figure 7:**
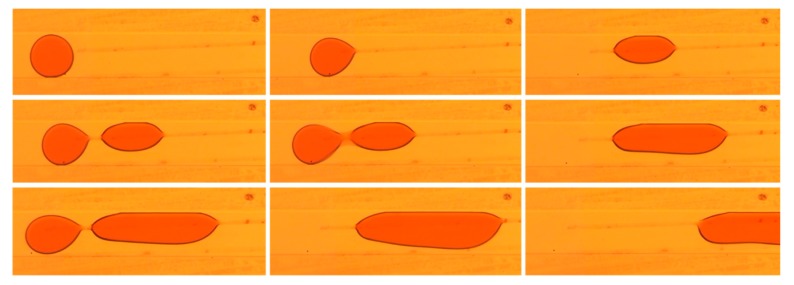
Coalescence and steering of disc-shaped droplets along a narrow band of GO with a width of 10 µm. The width of the channel is 200 µm.

The third case illustrates the effect of patterning GO for coalescing droplets and directing the motion of the merged droplet to a specific path. The coupled functions of coalescence and steering allow the construction of microfluidic systems that provide controlled reaction networks with no dispersion between the steered droplets [28]. A narrow line of GO with 10 µm width is patterned along the channel at a slight angle from the center of the channel and disc-shaped droplets are generated in a flow focusing device. The mechanism of trapping, merging, and transporting the merged droplet is demonstrated in Figure 7 for three droplets. The first droplet initially gets trapped as it adheres over the GO line and deforms as the forces of adhesion from the GO surface, the surface tension of the droplet, and the viscous drag from the continuous phase came into balance. When the second droplet gets in contact with the GO line, it experiences the same force balancing process and approaches the trapped droplet as it deformed in shape. The trapped droplets then collided and merged in a similar procedure as discussed above. The merging process repeated with the next droplet, at this stage the increasing viscous drag force competed with the GO surface forces resulting in the movement of the merged droplet along the GO line in the microchannel.

## 4. Conclusions

In this paper we present a new approach for droplet coalescence in microchannels by patterning the surface energy of a hydrophobic substrate with GO using standard microfabrication techniques. This approach is based on selective patterning of the surface energy of a hydrophobic surface for trapping dispersed droplets prior to their fusion. The GO patterned films on the COC were found to be stable at high thermal stresses without evidence of peeling off or deformation. Three representative cases were illustrated to demonstrate the effectiveness of the method on the fusion of droplets exhibiting different flow regimes and having different initial diameters, as well as the effect of changing the size of the patterned surface area on the fusion process. The results showed that the coalescence process depends on initial wetting of the GO with a thin film of water molecules prior to the trapping step. In addition, simultaneous coalescence and transport of the droplets inside the channel was achievable by manipulating the viscous drag forces in relation to the surface adhesion induced by the GO pattern. We believe this method will be useful for various micro-scale processes such as liquid–liquid phase separation, micro-extraction, and reaction networks.
